# Network and structural analysis of quail mucins with expression pattern of mucin 1 and mucin 4 in the intestines of the Iraqi common quail (*Coturnix coturnix*)

**DOI:** 10.14202/vetworld.2024.1227-1237

**Published:** 2024-06-08

**Authors:** Hazem Almhanna, Aqeel Mohsin Mahdi AL-Mahmodi, Abdulrazzaq B Kadhim, aArun H. S. Kumar

**Affiliations:** 1 Department of Anatomy and Histology, College of Veterinary Medicine, University of Al-Qadisiyah, Al Diwaniyah, Iraq; 2 Department of Anatomy, Histology and Embryology, Faculty of Veterinary Medicine, University of Kufa, Kufa, Iraq; 3 Department of Veterinary Biosciences, Stemcology, School of Veterinary Medicine, University College Dublin, Belfield, Dublin, Ireland

**Keywords:** gastrointestinal tract and network analysis, gene expression, mucin, quail

## Abstract

**Background and Aim:**

In avian and other species, mucins (MUCs) play a crucial role in the gastrointestinal tract (GIT), and constitute a large group of O-glycosylated glycoproteins, are glycoconjugate proteins. MUCs present in two forms: (1) membrane-attached on cell surfaces to repel external threats and (2) detachable, gel-forming proteins in the soluble form. In quail GIT, the specific types of MUCs that are expressed remain largely unknown. We investigated the expression of MUC1 and MUC4 MUCs in the GIT of Iraqi common quails and conducted network and structural analyses of all known MUC types across quail breeds.

**Materials and Methods:**

Histological and gene expression analyses of MUC1 and MUC4 were conducted using fresh small intestine and large intestine samples from 10 quails. Using the STRING Database, Chimera software, and PrankWeb-ligand binding site prediction tool, network and structural analyses of all reported types of quail MUCs were conducted.

**Results:**

Most intestinal MUCs in quails were acidic, with few neutral MUCs detectable through Alcian blue and periodic acid-schiff stains. Acidic MUCs were more expressed in the duodenum, ileum, cecum, and colon, whereas neutral MUCs were more expressed in the jejunum. MUC1 and MUC4 messenger RNA expression was significantly higher in the jejunum and colon than in the duodenum and ileum. The analysis of the network revealed that MUC 1, 15, 16, and 24 formed homologous networks, while MUC 2, 4, 5, and 6 formed heterologous networks. Specific MUC combinations, including MUC5A-MUC6, MUC5A-MUC5B, and MUC5B-MUC6, show higher intermolecular hydrogen bond formation affinity. MUC15, MUC16, and MUC24 showed minimal interaction with other MUC types. Among the analyzed MUCs, MUC5B, and MUC6 had the highest probability for binding, while MUC2, MUC4, and MUC5A showed lower probabilities despite greater numbers of binding sites.

**Conclusion:**

This study’s results offer significant insights into quails’ MUCs’ composition, expression, network interactions, and binding sites, advancing knowledge of MUC-related processes in gastrointestinal physiology and their potential connection to gastrointestinal diseases.

## Introduction

Mucins (MUCs) are a key component of mucus that have a major function in lubrication (reducing friction between surfaces) [1], act as biological filters (to trap microbes) [2], and sustainably hydrate tissues (keeping them moist) [3]. MUCs constitute a large group of O-glycosylated glycoproteins and are glycoconjugate proteins [1]. MUCs present in two forms: (1) membrane-attached on cell surfaces to repel external threats and (2) detachable, gel-forming proteins in the soluble form [2, 3]. MUCs, with molecular weights that are quite large and constructed of serine- and threonine-rich tandem repeat structures, vary in length based on MUC type [4]. MUCs, of various types, govern numerous functions, such as immune protection, cell signaling, and body lubrication, by interacting with monosaccharides, polysaccharides, and proteoglycans [5, 6]. MUCs, including gel-forming ones [7, 8], are responsible for forming a protective mucosal barrier in the intestine, which prevents adherence of infectious agents to the intestinal epithelium [9–11].

Birds, such as other animals, have a susceptibility to parasitic and microbial infections in their digestive systems [4]. These include bacterial infections such as *Salmonella* species and *Escherichia coli* [12], or protozoan infections such as giardia, trichomonads, and coccidia [13, 14], as well as parasitic infestations by tapeworms and roundworms [15, 16]. MUCs in the gastrointestinal tract (GIT) are essential for defense against pathogens primarily through non-specific defense mechanisms such as innate immunity, with humoral immunity playing a secondary role [17]. Specifically, MUC1 and MUC4, which are highly O-glycosylated glycoproteins, have large molecular weight, are membrane-bound [18, 19], are reported to be highly expressed in the colon of the human [20] and the GIT of pigs [21]. In various cancers, the contrasting expression and molecular alterations of MUC1 and MUC4 have been noted as promising prognostic indicators [20, 22–25]. MUC1 and MUC4 MUCs, linked to oncogenic roles in human tumor initiation and progression in various body locations, have expression and signaling pathways implicated in this process [26]. Detailed expression information for MUCs contributing to quail GIT health and functionality is unavailable [27].

This study examined MUC1 and MUC4 expression in various sections of the small and large intestines of the Iraqi common quail (*Coturnix coturnix*). To assess the biological significance and understand the functional mechanisms of all reported types of MUCs in various quail breeds, extensive network, and structural analyses were carried out.

## Materials and Methods

### Ethical approval

Healthy Iraqi common quail birds, approximately 8 weeks old, were humanely euthanized and killed by cervical dislocation under the Animal Ethics Guidelines of the University of Al-Qadisiyah’s College of Veterinary Medicine (Approval Ref. No. 1890).

### Study period and location

This study was conducted from December 10^th^, 2022 to November 1^st^, 2023. It was conducted at the Laboratory of the College of Veterinary Medicine, University of Al-Qadisiyah.

### Samples and study design

Ten quails (8–10 weeks, 160–200 g) were killed, and their duodenum, jejunum, ileum, cecum, and colon were harvested for histological examination and gene expression analysis. Two groups received the specimens. That group of samples was stored in TRIZOLe (SRCr Green-Zol reagent, Scientific Researcher Co. Ltd, Iraq) at −20°C for gene expression analysis using a quantitative polymerase chain reaction (qPCR) study. The other group was fixed in 10% neutral buffered formalin (100 mL formalin of 37–40% stock solution), 900 mL distilled water, 9 g of NaCl, and 12 g of Na_2_HPO_4_ (dibasic/anhydrous) at room temperature (25°C) for routine histopathological tissue processing and special staining procedures. Hematoxylin and eosin (H&E) distinguished the tissue structures of small and large intestines, while, periodic acid-schiff (PAS) with alcian blue identified their respective carbohydrate types.

### Histological analysis

Quail specimens were fixed in 10% neutral buffered formalin for 48 h, followed by routine processing, staining with H&E, alcian blue, and then with PAS. For 48 h, the tissue specimens stayed in 10% formalin before proceeding with histopathological processing, H&E, and a combination of PAS and alcian blue stains were used as described earlier [28, 29]. Representative images of stained tissue sections were captured using a light microscope (Olympus, Japan) at magnifications of 4×, 10×, 20×, and 40×.

### RNA extraction and complementary (cDNA) synthesis

The Accuzol® reagent kit (Bioneer, Korea) was used to extract total messenger RNA (mRNA) from the small and large intestines of quails. 200 mg of intestinal tissue for each part was weighed into separate 1.5 mL Eppendorf tubes (Jiangxi, China), and then 200 μL of chloroform each was added, mixed, and incubated on ice for 5 min. The supernatant was collected after centrifuging the tissues at 14,000× *g* (4°C) for 15 min. 500 μL of isopropanol was added, mixed, and incubated for 10 min at 4°C. The samples were centrifuged once more at 14,000× *g* and 4°C for 10 min. 1 mL 80% ethanol was added, vortexed, and centrifuged at 14,000× *g*, 4°C for 10 min after removing the supernatant. The pellet was removed and left to air dry in the Eppendorf tubes, discarding the supernatant. 50 μL diethyl pyrocarbonate water was added to and stored at −20°C for pelleted RNA. Each sample’s RNA concentration was measured using a Nanodrop spectrophotometer (Thermo Scientific, USA). The samples were treated with a DNase I enzyme kit (Promega, USA), according to the manufacturer’s instructions. Using the DiaStar™ OneStep reverse transcription PCR (RT-PCR) kit (China), total RNA was converted into cDNA following the manufacturer’s instructions and thermocycler conditions. Subsequently, the cDNA concentrations were normalized and stored at −20°C until use.

### Real-time RT-qPCR

**Figure-1:**
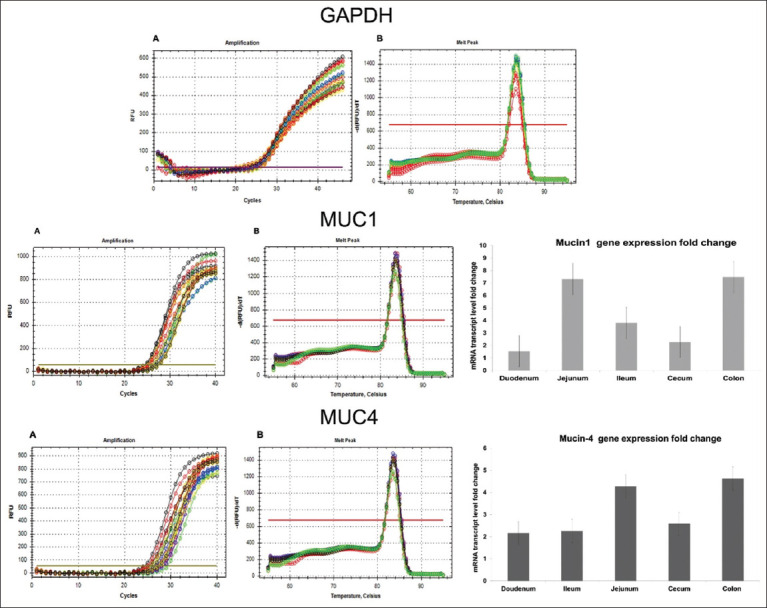
(a) The RT-qPCR amplification plots of gene and (b) PCR melting peak of the GAPDH MUC1 and MUC4 gene of the different regions of the small and large intestine of quail. The observable threshold cycle numbers of expression were specified and differences between regions of the intestine are shown. The bar graph illustrates the level mRNA expressions of the MUC1 and MUC4 in the duodenum, jejunum, ileum, cecum, and colon. RT-qPCR=Quantitative reverse transcription polymerase chain reaction, GAPDH=Glyceraldehyde-3-phosphate dehydrogenase, MUC=Mucin, mRNA=Messenger RNA.

RT-qPCR technique was applied for identifying the levels of MUC1, and MUC4 gene expression using the real-time PCR system (BioRad, USA). Following primers were used in this study; *Coturnix japonica* (CJ) glyceraldehyde-3-phosphate dehydrogenase (GAPDH) (gene code: XM_015873412.2) (GAPDH housekeeping gene), forward primer: TGCTGGCATTGCACTGAATG, Reverse: CACGGTTGCTGTATCCAAACTC, and CJ MUC1-like (LOC107317569) (MUC1), mRNA (gene code: XM_032448780.1) forward primer: TAATGCTGCCCCAATTGCTG, reverse primer: TGAGGTTGTATCCCAGTGCAG. CJ MUC4, cell surface associated (MUC4), mRNA, code: XM_032446547.1) forward primer: AATGCAAAGTGCCACAGCTG, reverse primer: TTGGTGTTCCTCCAAAACGC. The expression levels of GAPDH, MUC1, and MUC4 genes were measured using the SYBER Green dye qPCR master mix (Promega, USA) in accordance with the kit instructions (AccuPower™ 2×green Star qPCR master mix kit, Bioneer). In thermocycler, the initial denaturation was held at 50°C for an hour, followed by cycles of denaturation at 95°C for 20 s, annealing/extension at 60°C for 30 s, and a final melting temperature step set at 60°C–95°C for 0.5 s and repeated once. The amplifications and melting peaks showed a consistent curve without any non-specific product or amplification, with melting peaks ranging from 80°C to 88°C (Figure-1), thus providing a validation for our method used. RT-qPCR amplification plots of MUC1 and 4 genes of the duodenum, jejunum, ileum, cecum, and colon were accurately detected and threshold cycles (CT) numbers of expression of MUC1 and MUC4 were clearly noticed and ranged between CT 21.99 and CT 26.45 (Figure-1, Tables-1 and 2).

**Table-1:** This table presented values of gene expression of housekeeping gene and MUC1 which were analyzed using 2^∆CT^ method.

No.	Organs	CT (MUC1)	CT (GAPDH)	∆CT	Fold change (2^∆CT^)	Mean
1	Cecum	26.45	26.62	0.17	1.13	2.28
2	Cecum	24.72	26.61	1.89	3.72	
3	Cecum	25.14	26.13	0.99	1.98	
4	Colon	24.17	26.05	1.88	3.69	7.48
5	Colon	22.30	26.09	3.79	13.82	
6	Colon	24.34	26.64	2.30	4.92	
7	Duodenum	25.59	26.63	1.04	2.05	1.55
8	Duodenum	25.63	26.56	0.93	1.91	
9	Duodenum	26.74	26.21	-0.53	0.69	
10	Ileum	21.99	24.63	2.64	6.25	3.80
11	Ileum	25.11	26.92	1.81	3.50	
12	Ileum	24.83	25.56	0.73	1.65	
13	Jejunum	23.54	27.11	3.57	11.87	7.31
14	Jejunum	23.00	24.45	1.45	2.74	
15	Jejunum	22.96	25.83	2.87	7.32	

MUC=Mucins, GAPDH=Glyceraldehyde-3-phosphate dehydrogenase, CT=Cycle threshold

**Table-2:** This table presented values of gene expression of housekeeping gene and MUC4 which were analyzed using 2^∆CT^ method.

No.	Organs	CT (MUC4)	CT (GAPDH)	∆CT	Fold change (2^∆CT^)	Mean
1	Cecum	26.86	26.62	−0.24	0.84	2.59
2	Cecum	25.83	26.61	0.78	1.71	
3	Cecum	23.75	26.13	2.38	5.21	
4	Colon	23.23	26.05	2.82	7.07	4.63
5	Colon	24.11	26.09	1.98	3.95	
6	Colon	25.12	26.64	1.52	2.87	
7	Duodenum	25.13	26.63	1.50	2.84	2.16
8	Duodenum	25.67	26.56	0.89	1.86	
9	Duodenum	25.37	26.21	0.84	1.79	
10	Ileum	22.82	24.63	1.81	3.50	4.26
11	Ileum	25.11	26.92	1.81	3.51	
12	Ileum	23.03	25.56	2.53	5.77	
13	Jejunum	24.05	27.11	3.06	8.36	4.27
14	Jejunum	23.69	24.45	0.76	1.69	
15	Jejunum	24.36	25.83	1.47	2.78	

MUC=Mucins, GAPDH=Glyceraldehyde-3-phosphate dehydrogenase, CT=Cycle threshold

### Network and binding site analysis of quail MUCs

All the reported MUC sequences in quails were identified in the Uniprot database (https://www.uniprot.org/), and their 3D structure was generated using the AlphaFold (AF) (https://www.uniprot.org/) or Swiss homology modeling (HM) tools (https://swissmodel.expasy.org/) as reported previously. The network protein analysis of quail MUCs was conducted as reported before using the STRING Database (https://string-db.org), to observe its functional protein-protein interactions. The 3D structure of the quail MUC’s in Protein Data Bank format was imported onto the Chimera software (University of California, San Francisco, USA) and the number of hydrogen bonds (H-bond) formed between them at 10 Armstrong (10A) distance was evaluated. A heatmap was generated to identify high-affinity H-bond interactions between different MUC combinations. Using the PrankWeb: Ligand Binding Site Prediction tool (https://prankweb.cz/), the quail MUC’s binding sites were identified.

### Statistical analysis

The MUC1 and MUC4 gene expression levels were determined through RT-qPCR and the 2^∆CT^ method [30, 31], with significance assessed through one-way analysis of variance analysis using Statistical Package for the Social Sciences version 23.0 (IBM Corp., NY, USA) at a level of p ≤ 0.05.

## Results

### Histological assessment of quail intestines

**Figure-2:**
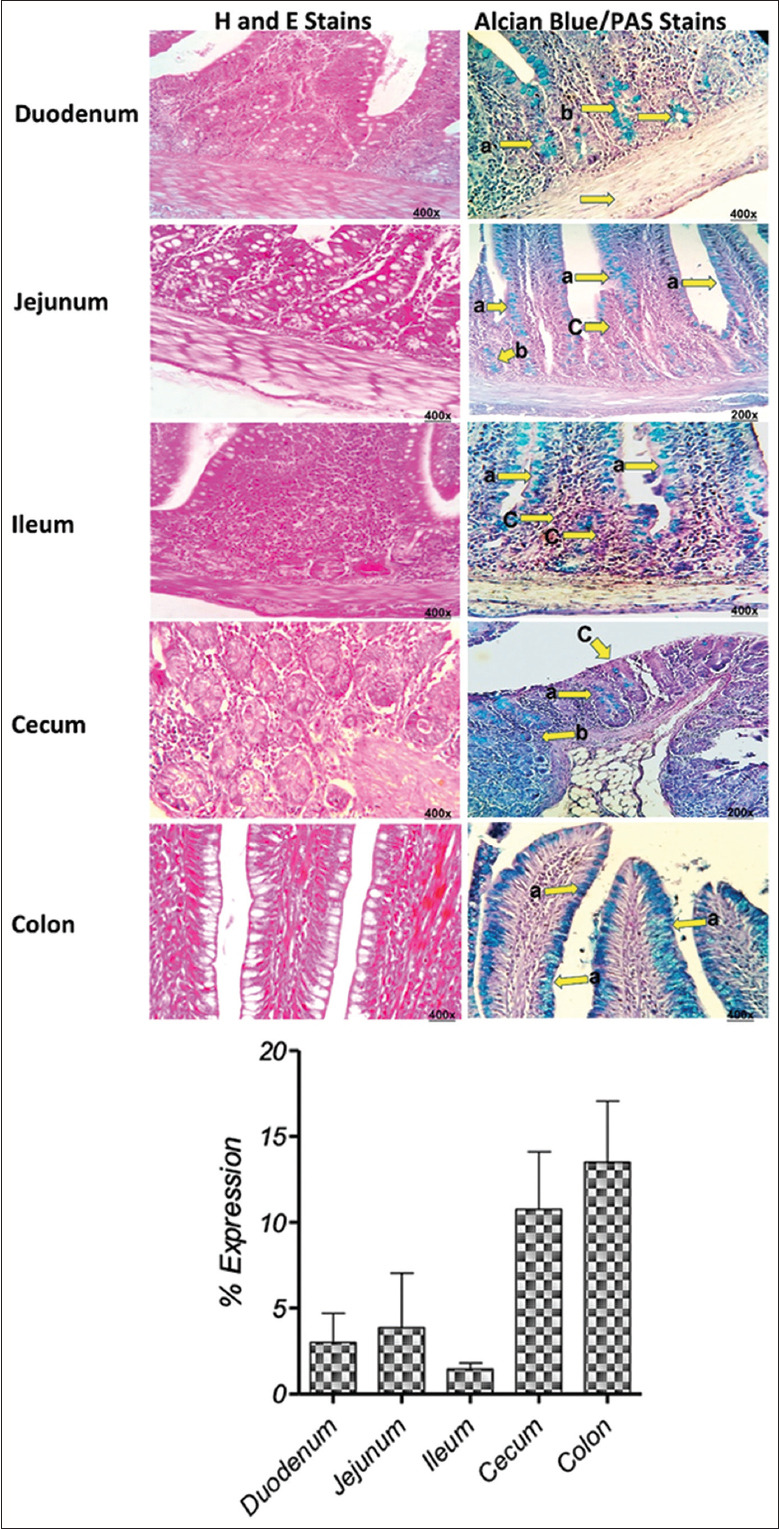
Histology of quail intestines. Representative histology sections of different parts of the small and large intestine of quail stained with H&E, and a combination alcian blue/PAS stains are shown. Photographs are labelled with (a) epithelial tissue, and (b) intestinal glands were positive stained with alcian blue, whereas (c) epithelial tissue was stained with PAS stain (400× magnificence). The bar graphs show the semi-quantification of the alcian blue staining area. Data are shown as mean±SD of percent expression of alcian blue staining area in the histological section assessed in three independent histological section per tissue type. H&E=Hematoxylin and eosin, PAS=Periodic acid-schiff, SD=Standard deviation.

H&E stains revealed the typical architecture of the quail’s duodenum, jejunum, ileum, cecum, and colon, consisting of mucosa, submucosa, muscularis, and serosa layers. The structure of each part of the quail intestinal tract conformed to avian literature. The quail intestinal tract exhibits three distinct mucosal layers: (a) a simple columnar epithelium with goblet cells on the basement membrane, (b) an extended lamina propria with intestinal glands, and (c) varying lymphocyte aggregations surrounded by loose connective tissue. In different regions of the intestine, the submucosa comprises diverse amounts of intestinal glands, fat globules, nerves, lymphatics, and blood vessels. The muscularis exhibited outer longitudinal and inner circular layers beneath a thin serosal cover (Figure-2). With alcian blue and PAS stains, both acidic and neutral MUCs can be observed at once. Acidic MUCs stained blue with alcian blue whereas neutral MUCs and glycogen turned pink/magenta with the PAS reaction. This combination of stains gives a comprehensive evaluation of the tissue’s MUC content. Epithelial MUCs in goblet cells and intestinal glands from the duodenum to colon were mainly stained blue (alcian blue), while PAS staining was scarcely seen. The staining pattern indicates that acidic MUCs are the predominant type in quails’ small and large intestines, with minimal occurrence of neutral MUCs. In Figure-2, neutral MUCs predominated in jejunum, while acidic MUCs were more prominent in duodenum, ileum, cecum, and colon. The acidic MUCs in cecum and colon were relatively more expressed than in the duodenum, jejunum, and ilium as shown by semi-quantified alcian blue staining (Figure-2). Ileum exhibited the least acidic MUC expression. The PAS staining intensity indicated a poor expression of neutral MUCs in the duodenum and colon of the quail intestine.

### Expression of selected MUC transcripts in quail intestines

During our investigation, we used RT-qPCR to detect MUC1 and 4 transcripts in different parts of the quail intestine. The levels of MUC1 and MUC4 mRNA were significantly amplified in both the small and large intestines (Figure-1 and Tables-1 and 2). The greatest levels of MUC1 and MUC4 mRNA were detected in the jejunum and colon with the least amounts in duodenum (for MUC1) and ilium (for MUC4) (Figure-1). In the jejunum and colon, MUC1 expression was markedly higher than MCU4. MUC4 expression was more prominent in the duodenum and cecum than MUC1 expression.

### Network analysis of quail MUCs

**Figure-3:**
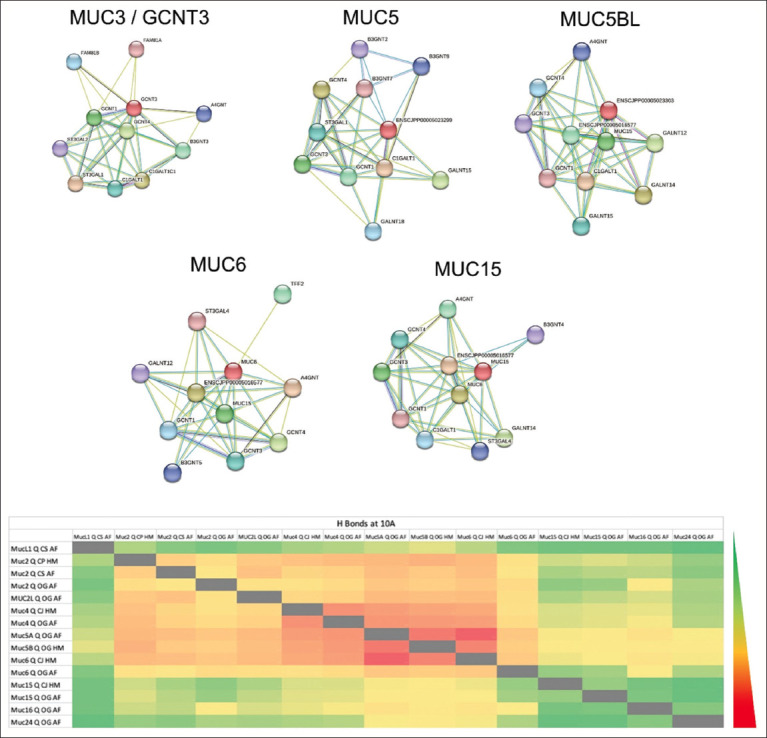
Network protein interaction of various quail mucin types in the STRING database. The heatmap represents the number of hydrogen bonds formed between each pair of quail mucins at 10 Armstrong evaluated in the Chimera software. 3D structure of 15 different quail-specific mucins were used in this analysis. OG=*Odontophorus gujanensis*, CJ=*Coturnix japonica*, CS=*Callipepla squamata*, AF=AlphaFold, HM=Homology Model. Scale: Red to green represents a high-to-low number of hydrogen bonds ranging from 25000 to 224 hydrogen bonds.

Uniprot database reveals 20 distinct MUCs particular to quails (Table-3). In quails, MUC24, MUC15, MUC16, MUC6, MUC5, MUC4, MUC2, MUC3, and MUC1 were the major identified MUC forms. Figure-3 illustrates the interactions of the five MUC types (MUC3, 5, 5BL, 6, and 15) found in the STITCH database (http://stitch.embl.de/). The predominant interactions among these networks in humans and quails were MUC-MUC, implying homologous associations (Figure-3, Tables-4 and 5). An in-depth examination of hydrogen bonding between each pair was conducted through the Chimera software to determine the biochemical interactions between different MUC combinations. Fifteen of the 20 quail-specific MUCs had distinct sequences allowing for AF or HM 3D structuring. Fifteen MUCs were investigated for intramolecular hydrogen bonding potential. The highest interactions were observed between the following combinations: MUC5A-MUC6, MUC5A-MUC5B, MUC5B-MUC6, MUC4-MUC6, MUC5A-MUC4, and MUC5B-MUC4. In Figure-3, MUC 15, 16, and 24 exhibited the least interaction with other MUC types. Based on our analysis of the MUC-MUC network, we identified two distinct classes of MUCs: those (MUC 2, 4, 5, and 6) that form heterogeneous networks and likely contribute to epithelial protection, and those (MUC 1, 15, 16, and 24) that demonstrate limited MUC-MUC interaction and may facilitate movement, transportation, and various cellular processes, including defense against pathogens, cell adhesion, differentiation, and inflammation.

**Table-3:** Various forms of MUCs expressed in different breeds of quails.

Uniprot ID	Gene	MUC type	Species
A0A7K9Z0E4	MUC15	MUC15	OG
A0A8C2TUH6	Mucin 15	MUC15	CJ
A0A7K9YXX0	MUC16	MUC16	OG
A0A7K9YTP5	Muc2_0	MUC2	OG
A0A8C2U6P4	MUC2	MUC2	CJ
A0A226MFN5	ASZ78_003200	MUC2	CS
A0A7K9YY45	CD164	MUC24	OG
A0A7K9YS25	Muc2l	MUC2l	OG
A0A8C2TP72	MUC4	MUC4	CJ
A0A7K9ZAF7	MUC4	MUC4	OG
A0A8C2TS61	MUC4	MUC4	CJ
A0A7K9YT94	Muc5ac_1	MUC5A	OG
A0A7K9YR83	MUC5B	MUC5B	OG
A0A8C2U825	LOC107314549	MUC5B	CJ
A0A226MF19	ASZ78_003201	MUC5B	CS
A0A8C2U8G1	MUC6	MUC6	CJ
A0A7K9YTQ5	Muc6	MUC6	OG
A0A226MUE7	ASZ78_012728	MUCL1	CS
A0A8C2SVR3	LOC107307211	MUCL3B	CJ
A0A8C2U4E5	LOC107314529	MUCL5B	CJ

OG=*Odontophorus gujanensis*, CJ=*Coturnix japonica*, CS=*Callipepla squamata*, MUC=Mucin

**Table-4:** Network analysis of human MUC1.

Predicted functional partner	Gene	No. of H bonds
Intercellular adhesion molecule 1	ICAM1	3010
Epidermal growth factor receptor	EGFR	3161
Catenin beta-1	CTNNB1	6275
Proto-oncogene tyrosine-protein kinase Src	SRC	4546
Galectin-3	LGALS3	4882
Mucin 5ac	MUC5AC	493
Cellular tumor antigen p53	TP53	3274
Estrogen receptor	ESR1	3161
Mucin 6	MUC6	9835
Mucin-4	MUC4	10986

MUC=Mucin

**Table-5:** Network analysis of quail MUC4.

Predicted functional partner	Gene	No. of H bonds
Receptor tyrosine-protein kinase erbB-2	ERBB2	9406
MUC16	MUC16	1993
Mucin 6	MUC6	15332
MUC1	MUC1	7874
Mucin 2	MUC20	6264
MUC13	MUC13	5239
Mucin-7	MUC7	2409
Receptor tyrosine-protein kinase erbB-3	ERBB3	9672
MUC15	MUC15	2755
Mucin-21	MUC21	10455

MUC=Mucin

### Binding site analysis of quail MUCs

**Figure-4:**
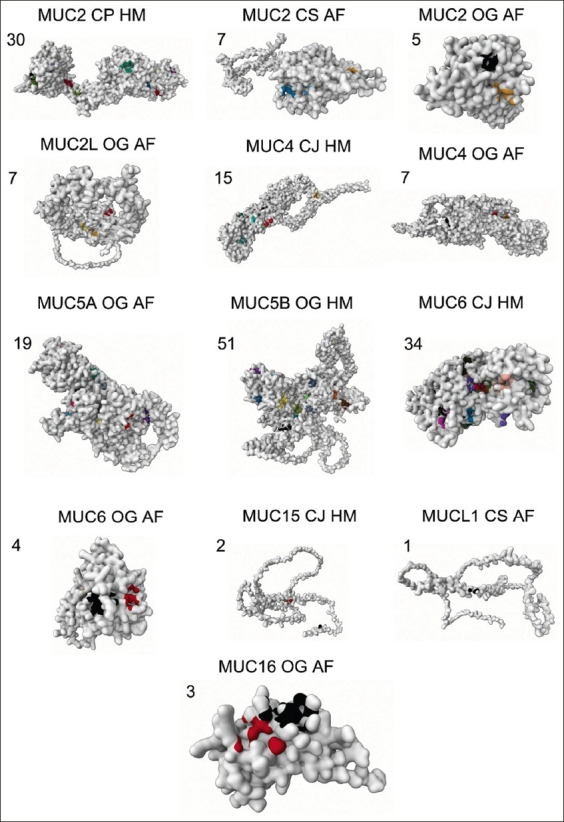
Binding site analysis of various quail mucin types. The values indicate the number of binding sites identified (represented in various colors). OG=*Odontophorus gujanensis*, CJ=*Coturnix japonica*, CS=*Callipepla squamata*, AF=AlphaFold, HM=Homology Model.

In inflammatory bowel disease (IBD), Crohn’s disease, ulcerative colitis, and colorectal cancers, changes to MUC production or the mucus layer composition are observed. Given the progress in comprehending the biological significance of MUCs in various gastrointestinal diseases, creating drugs that target specific MUCs is a promising prospect. Among the 15 quail-specific MUCs analyzed, MUC5B and MUC6 boasted the greatest number of high-probability (>0.8) binding sites as revealed in Figure-4 and Table-6. MUC 2, 4, and 5A, although they had more binding sites (>10 binding sites), their probability scores were lower (<0.6), suggesting their poor target ability or weaker interactions. MUC24 did not have any binding sites. All other MUCs (MUC 1, 15, and 16) with few binding sites (<3) and low probability scores (<0.1) displayed weak affinity interactions in our analysis. The sequence of the high affinity binding sites of MUC6 (A_1099 A_1102 A_1103 A_1106 A_1109 A_1110 A_1113 A_1121 A_1122 A_1127 A_1128 A_1129 A_1131 A_1156 A_1157 A_1158 A_1159 A_1160 A_1162 A_1172 A_1173 A_1174 A_1175 A_959 A_960 A_961 A_962 A_963 A_965 A_979 A_981 A_985 A_987), MUC5B (A_1123 A_1124 A_1127 A_1128 A_1136 A_1143 A_1146 A_1148 A_1150 A_1151 A_1156 A_1158 A_1161 A_1166 A_1167 A_1168 A_1177 A_1179 A_1182 A_1197 A_22 A_24 A_25 A_957 A_958 A_959 A_960 A_961 A_966 A_976 A_977 A_978), and MUC2 (A_1088 A_1089 A_1093 A_1101 A_1108 A_1110 A_1112 A_1115 A_1125 A_1126 A_1131 A_1132 A_1133 A_1142 A_1144 A_1147 A_921 A_922 A_923 A_924 A_925 A_941) identified here can be helpful in development of MUC specific selective small molecules or antibodies.

**Table-6:** Summary of the binding pockets in various quail MUCs.

MUC type	Uniport ID	No. of binding pockets (probability range)
MUC2_Q_CP_HM*	A0A8C2U6P4	30 (0.001–0.636)
MUC2_Q_CS_AF	A0A226MFN5	7 (0.004–0.082)
MUC2_Q_OG_AF	A0A7K9YTP5	5 (0.001–0.127)
MUC2L_Q_OG_AF	A0A7K9YS25	7 (0.003–0.208)
MUC4_Q_CJ_HM*	A0A8C2TS61	15 (0.002–0.319)
MUC4_Q_OG_AF	A0A7K9ZAF7	7 (0.021–0.44)
MUC5A_Q_OG_AF*	A0A7K9YT94	19 (0.001–0.295)
MUC5B_Q_OG_HM*	A0A7K9YR83	51 (0.005–0.855)
MUC6_Q_CJ_HM*	A0A8C2U8G1	34 (0.015–0.924)
MUC6_Q_OG_AF	A0A7K9YTQ5	4 (0.018–0.525)
MUC15_Q_CJ_HM	A0A8C2TUH6	2 (0.005–0.052)
MUC16_Q_OG_AF	A0A7K9YXX0	3 (0.017–0.098)
MUCL1_Q_CS_AF	A0A226MUE7	1 (0.028)
MUC24_Q_OG_AF	A0A7K9YY45	None
MUC15_Q_OG_AF	A0A7K9Z0E4	None

OG=*Odontophorus gujanensis*, CJ=*Coturnix japonica*, CS=*Callipepla squamata*, AF=AlphaFold, HM=Homology Model, MUC=Mucin

## Discussion

The study’s findings reveal intricate details of intestinal histology, MUC expression, MUC network interactions, and MUC binding sites in quails. Mucus plays a key role in maintaining the right environment for microflora of the intestine, regulating nutrient transport of foods, and immune response in addition to preventing pathogen invasion [32, 33]. As MUCs are the main components of mucus [34, 35], in this study, we investigated the various types of MUCs reported in different breeds of quails and specifically evaluated the transcripts of two types of MUCs (MUC1 and MUC4) in the various regions of quail intestines. Our study distinctly classified MUCs into two groups based on their network interactions: high-affinity MUCs forming diverse networks and low-affinity MUCs functioning alone.

According to avian literature [36–39], the quail intestines display a typical layered structure of mucosa, submucosa, muscularis, and serosa. The intestinal architecture of the quail, as assessed histologically, is comparable to that of other avian species [40, 41]. In the quail intestine, acidic MUC types have been reported to be the major expression, consistent with previous findings by Wilkinson *et al*. [42]. Acidic MUCs were predominantly expressed in the duodenum, ileum, cecum, and colon, whereas neutral MUCs had higher expression in the jejunum. Acidic MUCs are considered to play a prominent role in the protective functions of the quail intestinal mucosa, while neutral MUCs may have specific functions in the jejunum [43]. The high levels of acidic MUCs, characterized by negatively charged sialic acid residues, higher glycosylation, and numerous sulfate and carboxyl groups, in the cecum and colon, justify their lubrication, protection, and moisture retention functions. This study noted an unexpectedly high neutral MUC expression in the jejunum, which warrants further investigation regarding its functional significance. In various diseases, shifts in the equilibrium between acidic and neutral MUCs have been detected [44, 45]. IBD in humans is characterized by a shift from neutral to acidic MUCs [46, 47]. Whether the ANM (Acid to neutral mucin) ratio can function as a reliable biomarker for inflammatory bowel disease (IBD) needs further investigation.

We identified 20 different types of MUC reported in various breeds of quails but focused our gene expression analysis using RT-qPCR on MUC1 and MUC4 transcripts in different regions of the quail intestine as these two are the major transmembrane MUCs types which constitute intestinal mucosal layer [48, 49]. MUC1 and MUC4 mRNAs are found in both the small and large intestine, suggesting their roles in various physiological processes. The jejunum and colon had greater MUC1 and MUC4 mRNA expression than other areas. These MUCs’ roles in the jejunum and colon may be specific and associated with their functions in cellular processes, signaling, and protection. Studying the gene expression of other MUC types, particularly focusing on the network analysis results showing MUC 5, 6, and 2’s major influence on quail MUC physiology, is recommended for future research. Network analysis of quail MUCs elucidated potential functional relationships and interplay among them. The interactions among MUC2, MUC4, MUC5, and MUC6 indicate the presence of diverse MUC networks. These networks form a protective barrier that shields the epithelium from pathogens. MUC1, MUC15, MUC16, and MUC24 displayed weak interaction propensities, suggesting unique functions outside MUC networks. The relevance of biomarkers for digestive disorders based on the ratio of heterogeneous to homogenous MUCs remains uncertain.

MUC types and other components of the mucus layer interact within heterogeneous MUC networks [50–52]. The interactions among various MUCs within these networks likely affect mucus’s viscosity, lubrication, and particle-trapping capabilities [53, 54]. Heterogeneous MUCs possess strong affinities for interactions with other MUCs whereas homogeneous MUCs have weak interactions, functioning primarily on their own or with specific molecules or receptors. Due to their lower affinity for interactions, these substances might exhibit more independence, flexibility, and pinpoint targeting, thereby impacting distinct cell signaling pathways or functions. The lack of selective tools for targeting heterogeneous versus homogenous MUCs limits our understanding of their differences. Fifteen quail-specific MUC binding sites have been identified for the purpose of selective targeting.

The analysis of quail MUC binding sites focused on discovering candidates for targeted drugs or therapeutic approaches. MUC5B and MUC6, the quail-specific MUCs with the most binding sites and highest probability scores, are promising targets for drug development. The MUC2, MUC4, and MUC5A MUCs may be harder to target than MUC1, MUC15, and MUC16, given their limited binding sites and lower probability scores. We report in this study a high affinity binding site sequence of MUC2, MUC5B, and MUC6, which will be valuable in developing selective small molecules or antibodies against the binding site. MUC1 and MUC4 are reported to be involved in several important functions in the digestive system, including lubrication of the digestive tract, protection of the epithelial surface from mechanical and chemical damage, and regulation of the immune response [55–57]. Despite conflicting reports, this study’s network analysis implies significant roles for MUC5B, MUC6, and MUC2 in quails. Although MUC4 plays a significant role in quails due to its multiple low-affinity binding sites, the role of MUC1 is less significant due to the identification of only one very low-affinity binding site in quails [58–60].

## Conclusion

This study sheds light on the composition, expression, networking, and targetable aspects of quail intestine MUCs. Gaining insight into the function of MUCs in gastrointestinal processes and their significance for gastrointestinal disorders is pivotal for broadening our comprehension of mucosal defense, cellular mechanisms, and potential treatments. These results pave the way for future studies and may inform the creation of tailored interventions influencing MUC activities in birds and humans.

## Authors’ Contributions

HA: Designed the study and drafted and revised the manuscript. AMMA: Conducted the literature review, interpreted the data, and drafted the manuscript. ABK: Performed the laboratory work and explained the data of the gene expression in the manuscript. AHSK: Served as the project advisor, interpreted the data, and drafted and reviewed the manuscript. All authors have read, reviewed, and approved the final manuscript.
